# The complete chloroplast genome sequence of wild banana, *Musa balbisiana* variety ’Pisang Klutuk Wulung’ (Musaceae)

**DOI:** 10.1080/23802359.2018.1462123

**Published:** 2018-04-12

**Authors:** Ying-Feng Niu, Cheng-Wen Gao, Jin Liu

**Affiliations:** Yunnan Institute of Tropical Crops, Xishuangbanna, China

**Keywords:** *Musa balbisiana*, wild banana, chloroplast genome

## Abstract

*Musa balbisiana* is a wild-type species of banana, endemic to Southern China, Eastern South Asia, and Northern Southeast Asia. The *M. balbisiana* variety ’Pisang Klutuk Wulung’ is one of the possible ancestral parents of modern cultivated bananas, but its wild populations are now in decline. In this study, we report the complete chloroplast genome sequence of wild banana, *M. balbisiana* diploid variety ’Pisang Klutuk Wulung’. The *M. balbisiana* chloroplast genome is found to be 169,458 bp in length and has a base composition of A (31.44%), G (18.16%), C (18.61%), and T (31.79%). The genome contained two short inverted repeat (IRa and IRb) regions (35,084 bp), which were separated by a large single copy (LSC) region (87,805 bp) and a small single copy (SSC) region (11,485 bp). The genome encodes 113 unique genes, including 79 protein-coding genes, 30 transfer RNA (tRNA) genes, and four ribosomal RNA (rRNA) genes. Further, phylogenetic analysis suggested that *M. balbisiana* is closely related to the species of *M. textilis*. This complete chloroplast genome will provide valuable information for the development of DNA markers for future population and conservation studies of *M. balbisiana*.

*Musa balbisiana* is a wild-type species of banana native to Southern China, Eastern South Asia, and Northern Southeast Asia. The *M. balbisiana* variety ’Pisang Klutuk Wulung’, which has been shown to have very strong partial resistance to black leaf streak virus is one of the possible ancestral parents of modern cultivated bananas (Davey et al. 2013), along with *Musa acuminata*. However, with the decrease in the area of tropical rain forest and the lack of efficient conservation efforts, the original living environment of *M. balbisiana* was destroyed, the number of wild populations has fallen sharply, wild germplasm resources are seriously threatened.

The chloroplast genome has a wide range of uses in plant biology, such as the utility for phylogenetic inference and species identification(Sugiura et al. 1995). In this study, we report and characterize the complete chloroplast genome of *M. balbisiana* variety ’Pisang Klutuk Wulung’.

*Musa balbisiana* diploid variety ‘Pisang Klutuk Wulung’ was obtained from CIRAD (Guadeloupe) as rooted plants. Plants were maintained in a greenhouse. Genome sequencing was performed using Roche/454, sequencing libraries were prepared by the GS Titanium library preparation kit (454 Life Sciences, a Rochecompany, Branford, USA). The chloroplast genome was assembled using CLC Genomic Workbench v3.6 (http://www.clcbio.com). The genes in the chloroplast genome were predicted using the DOGMA program (Wyman et al. 2004). A circular map of *M. balbisiana* chloroplast genome was subsequently drawn using web-based program, OGDraw (Lohse et al. 2013). The complete chloroplast genome sequence together with gene annotations were submitted to the GenBank with the accession number of MH048658.

The complete chloroplast of *M. balbisiana* has a total length of 169,458 bp, which harbors a typical genome structure same as other plants (Sato et al. 1999; Ravi et al. 2008). The size of the *M. balbisiana* chloroplast genome is smaller than *M. acuminate*, larger than *M. textilis* and *M. itinerans*. In detail, the complete chloroplast genome of *M. balbisiana* is composed of a large single copy (LSC) region (87,805 bp), a small single copy (SSC) region (11,485 bp), and two short inverted repeat (IRa and IRb) regions (35,084 bp). The base composition of the circular chloroplast genome is A (31.44%), G (18.16%), C (18.61%), and T (31.79%). GC content of 36.77% for the whole *M. balbisiana* chloroplast genome. The *M. balbisiana* chloroplast genome encodes a total of 113 unique genes, including 79 protein-coding genes, 30 transfer RNA genes, and four ribosomal RNA genes. The genes repeated in inverted repeat regions comprised of eight tRNA, four rRNA genes, and 11 protein-coding genes. The introns were detected in 17 genes, of which ycf3 and clpP had two introns each, whereas the rest possessed only a single intron each.

To examine the phylogenetic position of *M. balbisiana*, eight selected complete plastome sequences were aligned using MAFFT program version 5.0 (Katoh and Standley 2013). The maximum likelihood phylogenetic was performed using MEGA7 (Kumar et al. 2016) (Figure 1). A bootstrap analysis was performed on the resulting phylogenetic tree, and values were obtained after 1000 replications. The result shows that *M. balbisiana* is close to *M. textilis*.

**Figure 1.**
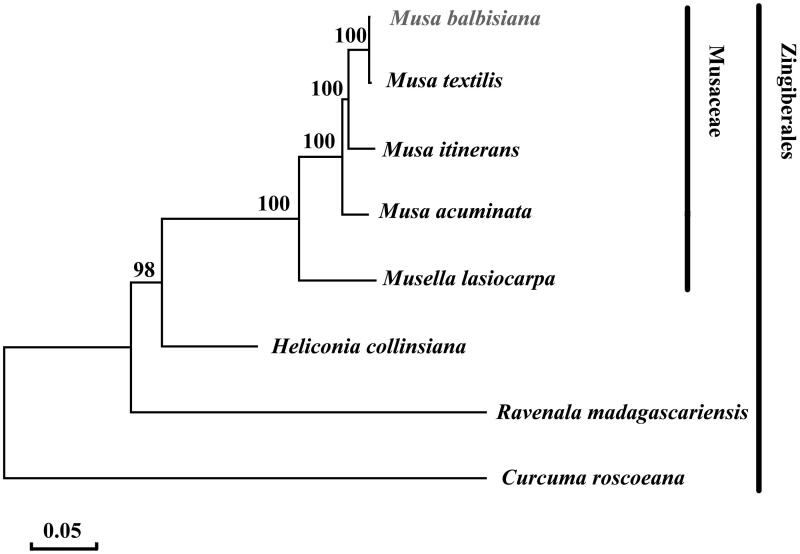
Maximum likelihood phylogenetic tree of *Musa balbisiana* with 7 species in the order Zingiberales based on complete chloroplast genome sequences. Numbers in the nodes are bootstrap values from 1000 replicates. Chloroplast genome accession number used in this phylogeny analysis: *Musa balbisiana* (MH_048658), *Musa textilis* (NC_022926), *Musa itinerans* (NC_035723), *Musa acuminata* (HF_677508), *Musella lasiocarpa* (NC_035637), *Heliconia collinsiana* (NC_020362), *Ravenala madagascariensis* (NC_022927), *Curcuma roscoeana* (NC_022928).

This newly sequenced chloroplast genome sequence will provide new genetic resource for studies of wild banana. Moreover, this study will be useful for further conservation of the wild banana.
